# Spinal Cord Stimulation in Chronic Low Back Pain Syndrome: Mechanisms of Modulation, Technical Features and Clinical Application

**DOI:** 10.3390/healthcare10101953

**Published:** 2022-10-06

**Authors:** Giusy Guzzi, Attilio Della Torre, Domenico La Torre, Giorgio Volpentesta, Carmelino Angelo Stroscio, Angelo Lavano, Federico Longhini

**Affiliations:** 1 Neurosurgery Department, “Mater Domini” Hospital, Magna Graecia University, 88100 Catanzaro, Italy; 2 Anesthesia and Intensive Care Unit, Department of Medical and Surgical Sciences, Magna Graecia University, 88100 Catanzaro, Italy

**Keywords:** Chronic Low Back Pain, Spinal Cord Stimulation, Pain

## Abstract

Chronic low-back pain (CLBP) is a common disease with several negative consequences on the quality of life, work and activity ability and increased costs to the health-care system. When pharmacological, psychological, physical and occupational therapies or surgery fail to reduce CLBP, patients may be a candidate for Spinal Cord Stimulation (SCS). SCS consists of the transcutaneous or surgical implantation of different types of electrodes in the epidural space; electrodes are then connected to an Implanted Pulse Generator (IPG) that generates stimulating currents. Through spinal and supraspinal mechanisms based on the “gate control theory for pain transmission”, SCS reduces symptoms of CLBP in the almost totality of well-selected patients and its effect lasts up to eight years in around 75% of patients. However, the evidence in favor of SCS still remains weak, mainly due to poor trial methodology and design. This narrative review is mainly addressed to those professionals that may encounter patients with CLBP failing conventional treatments. For this reason, we report the mechanisms of pain relief during SCS, the technical features and some clinical considerations about the application of SCS in patients with CLBP.

## 1. Introduction

Pain is a subjective unpleasant sensory and emotional experience associated with actual or potential tissue damage, or described in terms of such damage [1]. Pain is defined as chronic when lasting at least three months or more over the normal time of healing [2,3]. Chronic pain is a major public health concern, that affects hundreds of millions of patients and it costs hundreds of billions of dollars in associated medical charges and lost productivity [4]. By altering the nerve activity through electrical or chemical stimulus targeted to specific neurological sites in the body, neuromodulation is increasingly used in patients with chronic pain of varying etiology [5]. 

Chronic low-back pain (CLBP) is very common. CLBP prevalence has been reported by several studies and it increases with the age of the population. In particular, CLBP affects up to 4.2% of patients aged between 24 and 39 years old, and its prevalence increased up to 20% in the more aged population [5]. In addition, it varies among different regions and countries. This has been associated with several factors like lifestyle, occupational activities and the income level of the country [5]. 

The presence of CLBP causes activity limitation, with negative consequences on the daily quality of life, the ability to work, and increased and costly demand on the health-care system [6]. Strategies to treat CLBP aim to improve daily functioning by reducing the disability [1]. When not eligible for surgery, the approach to CLBP includes drugs (i.e., non-steroidal anti-inflammatory drugs, opiates, antidepressant drugs, local anesthetics), combined with psychological, physical and occupational therapies. If these strategies fail, the physician may assess the indication to combine Spinal Cord Stimulation (SCS) with other treatments (i.e., occupational therapy, rehabilitation and medications).

SCS is a modern neuromodulation technique to treat and reduce CLBP. It consists of an implanted generator of pulsed electrical signals conveyed to a precise region of the spinal cord through electrodes [7]. SCS is widely used to treat different forms of CLBP, including failed back surgery syndrome (FBSS), complex regional pain syndrome, peripheral neuropathic pain, ischemic disease and residual pain after joint replacement [8,9,10].

Although several authors have recently published reviews about the use of neuromodulation in chronic pain relief [5,11,12,13], none of them has specifically focused on the application of SCS in CLBP. We have therefore designed this narrative review to provide the rationale and clinical guidance for those professionals, including physicians, involved in CLBP treatment who need to refer patients to specialized facilities for SCS implantation. In the attempt to provide a valuable tool, we describe the underlying mechanisms of pain relief of SCS, the technical features and the procedure of implantation; finally, we analyze the clinical indications of SCS in CLBP.

## 2. Mechanisms of Pain Relief during Spinal Cord Stimulation

SCS relies on neurophysiological and neurochemical mechanisms of action, based on the “gate control theory for pain transmission” [14]. Two neurophysiological mechanisms are involved: the spinal segmental and the suprasegmental mechanisms [15].

### 2.1. Spinal Segmental Mechanisms 

Painful stimulations are transmitted through the nociceptive afferent neurons to the dorsal root ganglia, and, at the end, to the superficial (I/II) and deep (V) laminae, where they are modulated before being dispatched to the supraspinal centers. The dorsal column of the spinal cord contains large diameter fibers, carrying highly specific and detailed cutaneous and proprioceptive afferences, which do participate to the gate control of pain. Small diameter fibers (Aδ and C) ascend as a spinothalamic tract in the antero-lateral column and they carry the nociceptive stimuli to the supraspinal centers. In addition, the dorsal horn works as a relay station and integration site for nociceptive signals before ascending the pain control pathways [16,17]. 

### 2.2. Suprasegmental Mechanisms

SCS also interferes with the processing of the nociceptive signal through the lateral spinothalamic tract, modulating supraspinal brain centers such as the ventral posterior nucleus of the thalamus, somatosensory cortex, cingulate cortex, and insula [18,19]. Orthodromically, SCS can depolarize Aβ fibers in the cranial direction, therefore controlling the supraspinal centers like the cuneate and the gracile nuclei [16,17]. After supraspinal integration of the signal, descending feedback loops originating from the locus coeruleus [20], the nucleus raphe magnus [21] and the rostral ventromedial medulla [22] can modulate and control the spinal nociceptive signal at the “spinal gate” through both serotonergic and noradrenergic projections to the dorsal horn [16,17]. 

Concerning neurochemical mechanisms of action, SCS modulates neurochemicals such as gamma-aminobutyric acid (GABA) [23], serotonin [24], acetylcholine and norepinephrine [25,26]. In animal models, SCS was shown to increase the intraspinal release of GABA [27,28] and attenuate the excitatory response of glutamate and aspartate [29]. In addition, it has also been demonstrated that the most important type of GABA receptors involved in the inhibition of the nociceptive stimulation are type “b”, opening the possibility of intrathecal administration of sub effective doses of baclofen to enhance SCS analgesia [30].

SCS also increases the release of serotonin and substance P [24] and the expression and synthesis of dynorphin, and enkephalin within the dorsal horn of the spinal cord [31]. Of note, SCS decreases neuronal excitability and spinal pain transmission also by activating the 5-HT2A, 5-HT3 and 5-HT4 receptors [32].

Finally, SCS analgesia is also promoted by modulation of the cholinergic and adrenergic neurotransmission, by releasing acetylcholine and noradrenaline in the dorsal horn of the spinal cord [25,26].

## 3. Technical Features

### 3.1. Stimulation Waveform

The first application of SCS has been described in 1967 by Shealy et al. [33], now known as “conventional SCS”. A vitallium covered 3-4 mm electrode was surgically implanted after D2-D3 laminectomy and the patient reported the benefit of pain relief. Unfortunately, the patients died after 1.5 days from unexpected subacute endocarditis complicated by an embolism of the left side of the brain [33]. Conventional SCS consists in a tonic electrical stimulation of large Aβ sensory fibers with a moderate frequency range between 40 to 60 Hz. At this frequency, the stimulation produces paresthesia with the aim to cover the pain of the interested area. In particular, the excitation of Aβ fibers inhibits the neurons of the dorsal horn intended for nociception and its transmission to the supraspinal centers [16]. Settings of the stimulator (including amplitude, pulse width, frequency, stimulation configuration) are regulated to overlap the painful area with paresthesia [34]. For this reason, conventional SCS is also called paresthesia-based SCS [35].

Thereafter, the type of stimulation has evolved to a “burst-SCS”. This approach consists of intermittent bursts of electrical pulses (five pulses at 500 Hz, delivered 40 times per second) to mimic thalamic bursting within the nervous system. Compared to the conventional SCS, burst-SCS activates also the dorsal anterior cingulate and the dorsolateral prefrontal cortex [36], which mediate pain-related affect and attention [36,37] and the medial thalamic activity [38]. As a clinical advantage, burst-SCS improves pain relief by avoiding paresthesia, which is uncomfortable for the patient [36,39].

More recently, kilohertz-frequency SCS has been introduced in clinical practice. This latter is a tonic stimulation with a rate > 1kHz, up to 10Khz. This type of stimulation guarantees optimal (around 80%) pain relief without paresthesia [40]. In fact, this type of stimulation is programmed (i.e., pulse width and amplitude) to not produce paresthesia and to assure a subparesthetic stimulation [17]. Although a promising technique, there are conflicting data. Tiede et al. reported that high-frequency stimulation significantly improved the overall and back pain scores from baseline, and a higher responder rate, as compared to conventional SCS [41]. On the opposite, another recent study has demonstrated that pain relief is similar among different rates of stimulation (from 1 to 10 kHz) [42]. However, the body of evidence is more in favor of high-frequency rather than conventional low frequency stimulation [41,43,44,45,46].

### 3.2. Arrays and Electrodes

In addition to the type of electrical stimulation, the design of the lead is fundamental to optimizing pain relief. In principle, the electrical field should be shaped in order to optimize the stimulation. Increasing the number of electrodes improves the pain-to-paresthesia overlap and ameliorates pain relief [47,48]. At the beginning of its clinical application, SCS used intrathecal or subdural stimulation by means of arrays with four electrodes [48,49,50]. The development of cylindrical percutaneous arrays has increased the number of electrodes up to 16 [51]. Finally, paddle arrays may include from 16 to 32 electrodes, that are distributed in 2 to 5 columns, to improve the mediolateral resolution of the stimulation and to better focus the neuromodulation on the chosen dermatomes of the spinal cord [7,51].

With the continuous implementation of the SCS technique, the epidural stimulation has been preferred over the intrathecal or subdural one, due to reduced incidence of complications such as cerebrospinal fluid leakage and acute neurological deficit [7]. More recently, dedicated flexible lead arrays have been developed for subdural stimulation, to reduce the over mentioned complications, including damage to the spinal cord. The flexibility of these last leads allows the possibility of implantation directly near or on the spinal cord, and improves the targeting stimulation at lower amplitudes [52,53,54].

The choice of the type of electrode (i.e., percutaneous cylindrical versus paddle/plate electrodes) is an important issue in SCS implantation [55]. The choice of the technique of implantation varies among centers and it primarily depends on different protocols among centers [56,57,58]. A survey conducted in the United Kingdom aimed to assess the criteria of choice of the technique of SCS implementation [57]. Among the responders, 54% stated that the indication of implantation and the choice of the electrode were based on internal protocol and guidelines [57], which were in line with the key points suggested by the coeval European Pain Federation (EFIC) statements [59]. In a study by Kinfe et el., paddle electrodes were preferred in patients owing to a preoperative pain distribution requiring an electrode placement at a higher vertebral level to guarantee sufficient pain control [60]. The objective evidence to indicate which of the two electrodes is better, remains uncertain, although the percutaneous technique has the inner advantage to be minimally invasive [61]. Some authors have reported that paddle electrodes reduce the unwanted current spread and power consumption, while providing better coverage of the low back [58,62].

### 3.3. Pulse Generator

Implanted pulse generators (IPG) stimulate the spinal cord through precise extracellular voltages. However, the heterogeneity of impedance of electrodes may impair the ability to provide an optimal stimulation [7]. This drawback plays a major role in IPG with voltage-controlled stimulation, requiring specific and personalized adjustments [63]. Technical advances have created IPG based on current-applied stimulation. These systems are less affected by variations of impedance, guaranteeing a more stable stimulation of the spinal cord [51].

Another important technical advance in IPG are the multi-source systems. Most of the available IPG uses a single-source system, allowing the user to select the configuration for every single catheter as cathodes, anodes, or inactive [7]. In an attempt to improve the stimulation of the target areas, multi-source systems have been developed. An experimental model has proved that a multi-source system can target more central points of stimulation on the spinal cord, as compared to a single source system; this advantage may translate into a better paresthesia-pain overlap in patients with CLBP [64]. 

The innovations in rechargeable and longevity (up to 25 years) batteries also reduces the invasiveness and sizes of IPG [65,66]. Wireless systems have also been developed: a specifically design epidural passive electrode array, with a microprocessor receiver and an antenna, is implanted in the patient, whereas the pulse generator is not implanted, but worn by the patient [7]. The generator will transmit across the skin the parameters of the stimulation and the power to stimulate the spinal cord [7]. 

Another limitation for patients with SCS was the lack of possibility to perform magnetic resonance imaging (MRI), a fundamental exam in patients with CLBP. Of note, up to 84% of SCS-implanted patients could require at least one MRI exam within 5 years from the implantation [67]. The development and production of SCS systems with MRI-compatibility has also solved this difficulty in the last few years. 

Finally, SCS systems are generally implemented as “open-loop”. Although easier in their concept, they may be ineffective during body position changes. Indeed, when a patient changes his/her body position, the thoraco-lumbar spinal cord moves up to 3 mm in the anterior-posterior direction [68]. Since the strength of stimulation depends on the distance between the electrode and the neural target, little movements can invalidate the ability to properly stimulate the target area of the spinal cord, reducing the pain relief because of under-stimulation or inducing paresthesia for over-stimulation [69,70]. The variation of intensity of neuromodulation at the body position change has prompted the development of new devices which automatically adjust in real time the stimulation according to the position of the patient [70]. Another SCS system has been developed on the basis of the closed-loop technique. During SCS, evoked compound action potentials (ECAPs) are generated, representing the sum of the action potentials and providing a quantitative measure of neural recruitment in the spinal cord [71]. Therefore, when the amplitude of stimulation of the spinal cord varies, ECAPs accordingly modify as well. By using ECAPs as feedback and control signals, this new IPG modulates the amplitude of stimulation to guarantee optimal pain relief. Of note, the user is asked to define the reference ECAPs amplitude that the IPG targets in its closed-loop process [70]. Very recently, an algorithm for the optimization of SCS stimulation based on the Bayesian preference modeling has been proposed and validated in 5 patients with chronic (more than 1 year) traumatic spinal cord injury [72] and future data are expected from the ongoing randomized controlled trial [73]. However, this algorithm has not been so far tested and validated in patients with CLBP.

### 3.4. Procedure of Implantation

The procedure of SCS implantation typically implicates two consecutive stages [74]. 

**Figure 1:**
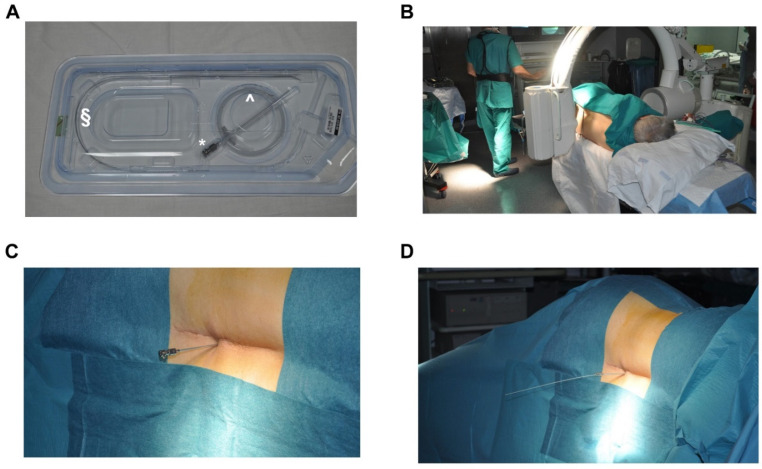
Technical features for electrode implantation.

**Figure 2:**
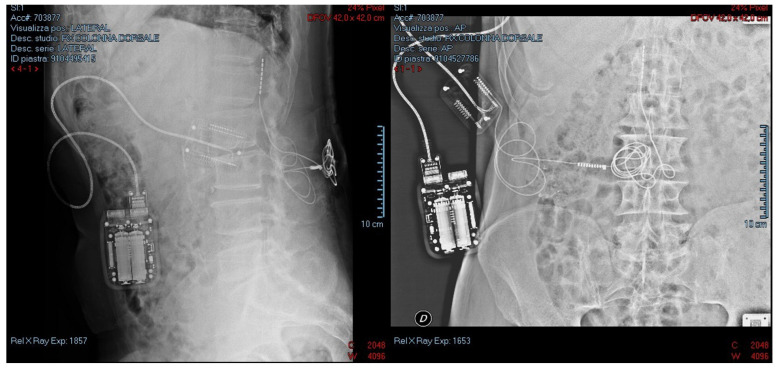
Anatomical position of the SCS.

The first one is a trial phase lasting between 3 to 10 days. As shown in Figure 1, the electrode arrays are dorsally implanted in the epidural space, a few levels above the segments of the spinal cord involved in painful symptoms [74]. The implantation of electrode arrays is performed through a Tuohy needle, under local anesthesia and with an X-ray check with a median or paramedian approach [4,75]. Electrodes are therefore connected to an external and temporary pulse generator to test the efficacy of the pain relief [74]. After the implantation procedure, a radiological check is also usually performed (See Figure 2). During this trial, stimulator parameters are regulated to optimize the treatment and control the pain [76]. In particular, the operator should adjust the amplitude, the pulse width, the frequency and the configuration of the stimulation. If the patient achieves pain relief equal to or greater than 50%, the treatment can be considered successful and the second phase of implantation is performed. The electrode arrays are therefore tunneled under the skin and connected to an implanted pulse generator (IPG), commonly placed in the posterior hip area [76]. The IPG is finally set with the parameters tested in the trial phase. 

The kit for SCS transcutaneous implantation is shown in panel A. The kit is constituted by a Tuohy needle (*), a wire guide (^) and the electrode (§). In panel B, the setting in the operating room is depicted. After the identification of the correct vertebral space (panel B), the Tuohy needle is inserted (panel C) and the electrode is positioned (panel D).

The figure shows the radiological check for the correct positioning of the electrode at L1 and of an external and temporary pulse generator for the trial phase.

Noteworthy, SCS electrode arrays could be also surgically implanted. This is the case of paddle- or plate-style electrode arrays, where laminotomy is required for their implantation [33,74,77]. Although more invasive, the surgical implantation of paddle leads has some potential advantages over the percutaneous technique, such as a more stable configuration reducing the risk of lead migration, the possibility to provide a unidirectional stimulation and a better clinical result at a 2-year follow-up when compared to percutaneous technique [7,55,56,58].

## 4. Clinical Considerations in Chronic Low Back Pain

The etiology of CLBP can be referred to as specific spinal cord lesions (such as radiculopathies or spinal stenosis) or not related to a spinal source. In the case of CLBP, physicians have to collect the clinical history and perform a detailed physical examination in order to understand the possible etiologies. If symptoms may be attributable to any spinal cause, magnetic resonance imaging or computed tomography of the spine is required to identify possible indications for pharmacological therapies, surgical treatments or physiotherapy. Noteworthy, the vast majority of patients with CLBP will not benefit from surgery, which remains indicated only in selected patients with functional disabilities or with refractory pain despite multiple nonsurgical attempted treatments [78]. If CLBP is not associated with spinal causes, reasons should be searched in other diseases, such as neoplasia, retroperitoneal cancers [79], inflammatory arthritis, infections [80], or other uncommon reported causes such as the engorgement of the epidural venous plexus secondary to inferior vena cava thrombosis [81].

The selection of patients is fundamental for the success of SCS treatment [82]. First of all, SCS should be considered within two years from the onset of symptoms, after the inefficiency of all conventional therapies [83]. Second, the presence of underlying psychiatric diseases, complete cognitive impairment, psychological comorbidities, or drug abuse preclude the possibility of SCS implantation [84]. However, in case of partial cognitive impairment, SCS may be considered and non-rechargeable should be preferred over rechargeable IPG [83]. Third, SCS should be also considered in case of neuropathic pain (i.e., failed back surgery syndrome, arachnoiditis, complex regional pain syndrome, causalgia, peripheral neuropathy, chronic radiculopathy), whereas in case of nociceptive symptoms or central neuropathic pain is not effective [83]. SCS is strongly recommended in case of [83]: failed back surgery syndrome in the absence of neurologic progression [85], axial low back pain [40] and complex regional pain syndrome [86]. SCS is also recommended in case of [83]: chronic refractory angina not controllable by maximal medical therapy, bypass surgery and percutaneous angioplasty of legs [87], peripheral artery disease or non-reconstructable critical leg ischemia [88]. 

As mentioned above, when a patient is a good candidate for SCS, an external and temporary stimulator is used to optimize the settings of the treatment to control the pain (first phase). If the patient achieves good pain relief (at least 50%), an IPG can be implanted after around 10 days (second phase). Noteworthy, a center for SCS implantations requires specific characteristics, such as trained personnel for diagnosis, indications, implantation and follow-up [83]. Of note, most of the centers for SCS implantation have a multidisciplinary team. Anesthesiologists and neurosurgeons are the most frequently involved; however, other professionals taking part in the team are nurses, occupation therapists, psychologists, psychiatrists, pharmacists and physiotherapists [57,89]. 

Once SCS is implanted, nearly half to 80% of the patients report immediate good pain relief with an indication of definitive implantation [10,90,91]. In these patients, SCS efficacy lasts up to 12 months [77,92] and in one study up to 24 months [10]. Some observational studies have also reported acceptable pain relief in 68% of the patients at a four-year follow-up [93], and in 52 to 74% of the patients at seven to eight years [94,95]. The efficacy of SCS may vanish over time for several reasons. The most frequent and important reasons are the migration of the lead [77,92], lead damage [96], infection of the insertion site [96] and the formation of scarred tissue around the lead [97,98,99,100].

Although the interest in SCS application to chronic pain is growing, to date large studies providing strong scientifically sound evidence are few [101,102]. From systematic reviews and meta-analysis [101,102], SCS seems to be a valid treatment when standard medical therapy fails to relieve painful conditions. However, definitive indications are difficult to be provided and future studies should address the effects of SCS on opioid reduction, functional improvement, and quality of life [102]. In addition, the reporting methods of the published literature and included populations are inhomogeneous, limiting the possibility to provide clear recommendations [101]. In fact, the quality of evidence that SCS is superior to re-operation (in case of failed back surgery syndrome) or conventional medical management has been recently defined as low-to-moderate [5]. For this reason, despite the growing literature body in this field, trials should improve their methodology to assure validity and replicability of the findings [101].

## 5. Conclusions

Among different techniques of neuromodulation, SCS is increasingly used to treat selected patients with CLBP resistant to other therapies (including drugs and physiotherapy). Technical advances in this field have improved the efficacy of pain relief and the treatment duration lasts up to eight years in around 75% of patients. More studies are however required to reinforce and to better define the current evidence in favor of SCS.

## Data Availability

Not applicable.
